# Job-Exposure Matrix: A Useful Tool for Incorporating Workplace Exposure Data Into Population Health Research and Practice

**DOI:** 10.3389/fepid.2022.857316

**Published:** 2022-04-26

**Authors:** Alexis Descatha, Marc Fadel, Grace Sembajwe, Susan Peters, Bradley A. Evanoff

**Affiliations:** ^1^Univ Angers, CHU Angers, Univ Rennes, Inserm, EHESP, Irset (Institut de recherche en santé, environnement et travail)-UMR_S 1085, SFR ICAT, Angers, France; ^2^CHU Angers, Poisoning Control Center-Clinical Data Center, Angers, France; ^3^Department of Occupational Medicine, Epidemiology and Prevention, Northwell Health, Hofstra University, New York, NY, United States; ^4^Institute for Risk Assessment Sciences, Utrecht University, Utrecht, Netherlands; ^5^Division of General Medical Sciences, Washington University School of Medicine, St. Louis, MO, United States

**Keywords:** public health, occupational exposure, job exposure matrix, JEM, epidemiology

## Abstract

Workplace exposures to physical, chemical, and psychosocial factors account for a large burden of chronic diseases. Obtaining useful estimates of current and past workplace exposures is challenging, particularly in large general population studies. Job-exposure matrices (JEMs) are a useful tool for exposure assessment, particularly when no individual level exposure data are available. A JEM provides a cross-tabulation of job titles (sometimes combined with industry) and estimated exposures to workers carrying out these jobs during different time periods. The major limitation of JEMs is that they do not account for individual variation in exposures within the same job. This limitation is offset by the advantages of low cost, wide applicability, lack of bias from self-reporting, and the ability to estimate exposures based on job titles when no other exposure data exist. There is growing use of JEMs in research examining the role of workplace exposures in the development of chronic diseases, and interest in their application to public health practice. This paper provides a scoping review of JEM use, some examples of JEMs, and brief guidance for the application of JEMs in epidemiological research. In conclusion, JEMs provide a useful tool for researchers and public health practitioners to estimate occupational exposures in large scale epidemiological studies relevant to many health conditions.

## Why Are Job-Exposure Matrices a Useful Tool for Public Health?

There is a significant global concern in preventing chronic diseases such as cardiovascular, respiratory, neurodegenerative and musculoskeletal diseases, cancer, as well as communicable diseases (including Sars-CoV-2) (1, 2). The multiplicity of disease determinants makes their prevention complex, especially since the relevant factors for many diseases occur throughout a long part of the life span. The totality of external exposures to which each individual is exposed from conception to death, including exposure resulting from environmental agents, socioeconomic conditions, lifestyle, and diet, is called the “exposome” (3). On a population level, identifying the complex relationships between multiple determinants of health is critical for improving population health (4). Among these health determinants, diseases resulting from specific workplace chemical, biological and physical exposures have been recognized for centuries. More recent work has recognized that a broad array of workplace exposures including chemical exposures, workplace physical activities, long working hours, and workplace psychosocial exposures are important determinants for many common diseases including cancer and musculoskeletal disorders (5, 6). For example, population level studies have estimated that 2–8% of cancers and over 20% of upper extremity musculoskeletal disorders may be attributable to workplace exposures (7, 8). However, most population health studies do not account for work exposures, and many public health datasets used for research contain little work information (9).

One reason for lack of inclusion of occupational exposures in most epidemiological studies is that workplace exposures are difficult to assess, and studies of occupational exposures are often limited to specific high-risk working populations, rather than focusing on common exposures across multiple jobs at a population level (10). Obtaining valid estimates of occupational exposures is an important objective for all epidemiological studies of working populations. Direct measurement of work exposures and observation of individual workers are precise and accurate, though they are expensive, time consuming, and difficult to apply in a general population setting (11, 12). Expert assessments allow assessors to incorporate general knowledge of the exposure field, but they are expensive and may miss some individual-level exposures only known to the workers themselves. Questionnaires are easier to administer to large populations, but self-reported exposures may be inaccurate, or biased by current or past health conditions (13).

## What Is a Job-Exposure Matrix?

Simply put, a job-exposure matrix (JEM) is a table that links job titles with indices of exposure to one or more work factors (14, 15). From the relatively simple collection of subject's job titles, JEMs make it possible to infer more complex information, by converting coded job titles into exposure estimates for epidemiological studies (16, 17). JEMs have been used for chemical exposure assessment for several decades, particularly for estimating past exposures and in populations without individual level exposure data (18, 19). The use of JEMs is an accepted method to estimate worker's exposure to chemical and other physical risk factors based on job titles, industry information, and population exposure data (14, 15). Many JEMs have been developed over the past decades (20), and are available for research and public health applications, allowing workplace health determinants to be included in the exposome approach (3).

## How Is a Job-Exposure Matrix Constructed?

JEMs are typically constructed based on expert assessment, self-reported exposures, or monitoring data, or by a combination of these methods. JEMs vary widely by type of exposures assessed, and by applied exposure metric, for instance by probability, frequency, duration, or level of exposure (21). Choices for these metrics are often driven by what data are available. By design, JEMs pool individual level exposure data at the level of job title or group of job titles, with the goal of creating low within-group variability and high between-group contrast of exposure estimates that differentiate job groups (22). JEM developers also seek good agreement between experts involved in their construction, good correlation with other methods of estimating work exposures, and the ability to reproduce known exposure-disease associations. The choice of a JEM for application depends on what exposures are relevant to a particular health outcome, the presumed time course of relevant exposures (past or recent) and the intended population under study. JEMs must also be linked to the job title coding system used in the population of interest. Different countries have different job coding systems, and while most can be linked through a “crosswalk” to ISCO (International Standardized Occupational Codes), there are different versions of ISCO codes, and transcoding of jobs from one system to another can introduce misclassification (23, 24). In some cases, activity sectors are important to consider with the International Standard Industrial Classification developed by United Nations (version 4 now) (25).

## Examples of Job-Exposure Matrices

Since JEMs were first described in 1983 (Box 1), there has been a steady increase in the number of published articles on JEMs. Five countries account for 50% of the publications on the subject (United States, France, Sweden, the Netherlands, and Denmark). Among the 1,208 articles, most papers were based on chemical JEMs (66%), and 13% were methodological papers. Publications on biomechanical and psychosocial exposure have grown in the last decade, though chemical JEMs are the most common. The main health disorders studied with JEMs are cancers (47%), respiratory diseases (18%) and musculoskeletal diseases (8%). There are many fields for development for future research, including physical hazard JEMs such as electromagnetic frequencies and noise (26, 27), and for biological hazards including COVID-19 (28–30). We highlight several JEMs below to illustrate their variety in construction and application.

Box 1 **Scoping review on JEM.**

A scoping review was conducted in September 2021 on the PubMed and Scopus databases, using several search terms for Job Exposure Matrix included in the title, abstract, or keywords. No exclusions were made. In total, 1,208 articles were found (including 980 in PubMed), with a first article in 1983 and over 80 papers in the last years, mostly coming from North America and Europe. The majority were original articles (*n* = 1,096), with only 60 reviews and 5 editorials. Where these papers originated from occupational and environmental journals (*n* = 746, 61.8%), 211 were clinical journals (mostly on cancer or respiratory topics, 17.4%), and 132 from other Public Health journals (10.9%).

### FINJEM

The Finnish job-exposure matrix (FINJEM) was constructed for exposure assessment in large registry-based studies (19, 31). The exposures in FINJEM cover major physical, chemical, microbiological, ergonomic, and psychosocial factors in the assessment period 1945–1997, divided into several subperiods. Exposures are described by the prevalence of exposure and the level of exposure among the exposed, both estimated mainly on continuous scales. FINJEM has been used for assessing occupational risk factors in many studies (32, 33) and there are several derivates from FINJEM (20).

### The Occupational Information Network (O^*^NET)

The Occupational Information network (O^*^NET) is a large, publicly available American dataset containing information on physical, psychological, and other job demands for more than 800 occupations (34). These estimates of job demands were created through expert opinion and from self-reported exposures by individual workers across different jobs. Job demands in O^*^NET are scored on ordinal scales with exposure-specific descriptive anchors, with jobs coded using the American standard occupational classification (SOC) job codes. Exposures from O^*^NET have been used in American population studies of a variety of conditions including asthma and musculoskeletal disorders (35, 36). O^*^NET has also been linked to job titles in the UK Biobank Study, and used to estimate work exposures relevant to SARS-CoV-2 and rotator cuff disorders in this large British cohort study (37).

### JEM Constances

JEM Constances is based on 27 different biomechanical factors and physical activities reported by ~35,000 active workers participating in a large French prospective cohort study (38). These self-reported exposures, based on four or five category scales, were pooled at the level of the job using the French Classification of Occupations (*Profession et Catégories Socioprofessionnelles* PCS 2003), and subsequently transcoded to ISCO version 2008 (24). For each exposure the JEM gives the distribution of exposures reported in >400 different jobs. This JEM has been used to examine associations between cumulative work exposures and the risk of Dupuytren's contractures and decreased physical abilities in later life (39, 40).

### SYN-JEM

Within the scope of the SYNERGY project, a large pooled analysis of lung cancer case-control studies (41, 42), exposure measurements have been collected throughout Europe and Canada (43). The quantitative JEM (SYN-JEM) was developed by modeling of individual measurement data, to assign exposures to five major lung carcinogens [asbestos, chromium, nickel, polycyclic aromatic hydrocarbons (PAH), and respirable crystalline silica (RCS)]. The exposure database allowing for such quantitative exposure assessment included 356 551 measurements from 19 countries. Measurements were distributed over the five agents as follows: RCS (42%), asbestos (20%), chromium (16%), nickel (15%), and PAH (7%). The measurement data cover the time period from 1951 to 2009. Mixed-effects models were developed including the personal occupational exposure measurements from Europe and Canada, as well as auxiliary information on job, year of sampling, region, an a priori exposure rating of each job (none, low, and high exposed), sampling and analytical methods, and sampling duration. The model outcomes were used to create SYN-JEM with a quantitative estimate of the level of exposure by job, year, and region (44, 45).

### Occupational Asthmagen JEM (OAsJEM)

In the context of the growing number of asthmagens and the importance of understanding of the etiological role of irritants, an occupational asthmagen JEM was developed in the late 1990's and recently updated (OAsJEM) (46, 47). A working group of three experts evaluated exposures for each 1988-ISCO job code into three categories: “high” (high probability of exposure and moderate-to-high intensity), “medium” (low-to-moderate probability or low intensity) and “unexposed”. The OAsJEM covers exposures to 30 sensitizers/irritants, including 12 newly recognized, classified into seven broad groups.

## What Are the Limitations of JEMS?

By assigning the same exposure to all workers in a job, JEMs reflect an “average” level of exposures, and cannot account for exposure heterogeneity among individual workers within the same job. This creates non-differential misclassification of estimated individual exposures, which differ within a job due to individual behaviors and other factors (48, 49). JEMs work best when there is large variability between the exposures in different jobs, and are less able to discriminate between groups of workers with similar exposures.

JEMs must also be used with caution given the limitations of their construction, based either on expert assessments or on data collected at the individual and aggregate level. The expertise and opinions of the experts influence the exposures assigned in the JEM and can result in exposure misclassification (50). Coding of occupations from job titles and industry can be time intensive, and can influence the exposures assigned to individual workers. Automated job coding systems exist for several national job coding systems, though such automated coding still need to be supplemented by manual coding (51).

## Why Use JEMS in Public Health Research and Practice?

In addition to the use of JEMs for studies specifically assessing associations between workplace exposure and related disorders, it is increasingly clear that public health risk factor models should include all relevant factors in the “exposome,” including work (4). Indeed, given the large amount of time that people spend at work, exposures and behaviors at the workplace should be considered when describing risk factors for future disease. There is increasing interest in assessing the role of workplace factors on chronic and acute diseases that have not been traditionally considered to be “occupational diseases.” For instance, a JEM was used to study the influence of workplace physical exposures on aging in the Copenhagen Aging and Midlife Biobank (52). In addition to the known risk factors of age, smoking, body mass index, hypertension, diabetes, and socioeconomic status, atrial fibrillation was also found to be independently associated with exposure to occupational psychosocial stress (assessed with a JEM), in a large study of the Swedish population (53). Another study used a JEM to assess lifting during pregnancy and found an increased risk of stillbirth among women with a prior fetal death who lifted over 200 kg/day at work (54). In the COVID crisis, several JEMS have been developed to estimate occupational exposures related to the risks of exposure and infection with SARS-CoV-2 (29, 30, 55). These examples illustrate the importance of considering occupational exposures in combined risk factor studies, and JEMs can offer efficient opportunities for public health researchers to incorporate workplace exposures into their analyses.

In addition to research applications, JEMs may play a role in risk factor surveillance and other public health activities, and in clinical management (56). JEMs could assist in the clinical care of workers, in return to work assessments, and in the worker's compensation or other social benefits process, by providing basic information on relevant exposures within different jobs. Because occupational diseases are often under-recognized, another practical application is using a JEM to screen for occupational exposures as part of health surveillance. By summarizing multiple exposures at a job level, JEMs may also assist policy-makers in setting priorities for hazards and controls at work, and assist occupational practitioners to target prevention efforts and direct the conduct of more precise exposure measures to particular jobs.

Several international research initiatives are working on improvements in the use of JEMs. For instance, the Exposome Project for Health and Occupational Research (EPHOR, www.ephor-project.eu) (57) and JEMINI (Job Exposure Matrix InterNatIonal) (58) initiatives are exploring the possibility of developing international JEMs that could be used across countries.

Finally, some networks of JEMs have been proposed. For instance, the Danish Occupational Cohort with eXposure data (DOC^*^X) contains measures of a wide variety of occupational exposures provided by JEMs (59). In addition to chemical, psychosocial, and biomechanical factors, a JEM covering lifestyle factors associated with both job/industry and health/disability has been developed including factors such as smoking, alcohol consumption, body mass index, and leisure-time physical activity (60).

## Conclusion

Given the many hours people spend at work and the risk of common diseases attributable to occupational exposures, including workplace physical activity and psychosocial factors, there are clear opportunities for improving risk factor models by incorporating workplace exposures. JEMs can be a powerful tool for exposure assessment in epidemiological studies, particularly in large-scale studies with limited occupational information. This useful tool should be used more widely outside the field of occupational disease epidemiology (Box 2, Table 1).

Box 2 **Practical guide for choosing a JEM.**

Several items need to be considered by researchers wishing to apply a JEM to their data.

First, is there is a relevant JEM that can be used?

**1. Is there an existing JEM on the exposure of interest?**

In addition to common research databases (Pubmed, Scopus, Web of Sciences), researchers can look at online repositories of available tools such as (https://occupationalexposuretools.net/inventory/.

**2. Is the JEM available?**

Although most JEMs were publicly funded, are intended to be used widely, and are available through public websites or from their developers, restrictions of use and availability should be checked.

**3. What job coding system is used?**

Job title information in the source data must be linked to the exposure data in the JEM. Ideally, the JEM uses the same job coding system as the source data – if these do not match, then transcoding of job titles will be needed to link to the JEM. Even with the same coding system, attention must be paid to which version of standard occupational codes was used, as job and industry codes change over time.

**4. Does the JEM contain relevant exposure metric(s)?**

JEMs vary widely on what exposure data are included, and at what level of detail; the hypothesis under study will drive the choice of the JEM. The analytic plan must consider which exposure(s) will be used, and how to define exposure levels (dichotomous or continuous) and exposure periods.Next, is the selected JEM appropriate for the research question?

**5. Be aware of a JEM's general limitations (and strengths)**

Because a JEM assigns the same exposure level to each worker in the same job, it will not account for individual exposure variation within a job. Inaccuracies in coding job titles may introduce additional misclassification. However, JEM exposure assignments are unbiased by disease condition, and by differential recall of exposures.

**6. Are the methods of JEM development and validation appropriate?**

Since every JEM might have specific limitations related to their development and validation, researchers should be aware of how the selected JEM was developed and how it has been used in other studies.

**7. Consider validation in the context of the proposed study**

Additional validation of a JEM may be possible within the context of a new population by checking known exposure-disease associations as positive and negative controls.

**Table 1:** Checklist for JEM application.

	**Yes/No/ to be checked**	**Comment**
Existing for exposure of interest?		
Available?		
Job coding applicable?		
JEM contain relevant exposure metric(s)?		
Aware of general limitations (and strengths)?		
Methods of development and validation are appropriate?		
Validation in the context?		

## Author Contributions

AD: conceptualization. AD, MF, and BE: writing original draft. SP and GS: review and editing. All authors have approved the final version submitted to the journal.

## Funding

AD was supported by public grants: REACTing Inserm (≪Mat-O-Covid project≫), ANRS | Maladies infectieuses émergentes since 2021), Regional public fund (TEC-TOP project, Pays-de-la-Loire Region, Angers Loire Métropole, Univ Angers/CHU Angers). Funding sources had no role in the study/manuscript.

## Conflict of Interest

The authors declare that the research was conducted in the absence of any commercial or financial relationships that could be construed as a potential conflict of interest.

## Publisher's Note

All claims expressed in this article are solely those of the authors and do not necessarily represent those of their affiliated organizations, or those of the publisher, the editors and the reviewers. Any product that may be evaluated in this article, or claim that may be made by its manufacturer, is not guaranteed or endorsed by the publisher.
